# Patient characteristics and treatment efficacy after switching to hypoxia-inducible factor-prolyl hydroxylase inhibitors from erythropoiesis-stimulating agents in non-dialysis-dependent chronic kidney disease patients: the Reach-J CKD cohort study

**DOI:** 10.1186/s12882-026-05036-3

**Published:** 2026-05-25

**Authors:** Kunihiro Yamagata, Tomohiro Ohigashi, Chie Saito, Hirayasu Kai, Ryoya Tsunoda, Reiko Okubo, Masahide Kondo, Haruka Ishii, Masami Inuzuka, Kenji Harada, Ichiei Narita, Hirokazu Okada, Takashi Wada, Shoichi Maruyama, Junichi Hoshino

**Affiliations:** 1 Department of Nephrology, University of Tsukuba, 1-1-1 Tennodai, Tsukuba, Ibaraki 305-8575 Japan; 2 Department of Biostatistics, University of Tsukuba, Tsukuba, Japan; 3 Department of Health Economics, University of Tsukuba, Tsukuba, Japan; 4 Medical Affairs Department, Kyowa Kirin Co., Ltd., Tokyo, Japan; 5 Division of Clinical Nephrology and Rheumatology, Niigata University Graduate School of Medical and Dental Sciences, Niigata, Japan; 6 Department of Nephrology, Saitama Medical University, Moroyama, Japan; 7 Department of Nephrology and Rheumatology, Kanazawa University, Kanazawa, Japan; 8 Department of Nephrology, Nagoya University, Nagoya, Japan; 9 Department of Nephrology, Tokyo Women’s Medical University, Tokyo, Japan

**Keywords:** HIF-PH inhibitors, Anemia in chronic kidney disease, Erythropoiesis-stimulating agents

## Abstract

**Background:**

Hypoxia-inducible factor-prolyl hydroxylase inhibitors (HIF-PHIs) are likely to be effective in patients with anemia in chronic kidney disease (CKD) who are resistant to treatment with erythropoiesis-stimulating agents (ESAs). However, few studies assess HIF-PHIs in patients with elevated ERI who do not yet require hemodialysis.

**Methods:**

This multicenter, prospective, observational study utilized data from the Reach-J study to explore the characteristics of Japanese patients with anemia in CKD (stage G3b–5) who switched from ESAs to HIF-PHIs. We also examined changes in hemoglobin levels following switching.

**Results:**

We assessed 447 patients who continued ESA treatment and 34 patients who switched from ESAs to HIF-PHIs (mean age: 74 years). Patients who switched had numerically higher mean erythropoietin resistance index (ERI) and numerically lower hemoglobin than those who continued ESA treatment. Mean hemoglobin levels increased following switching (9.9 g/dL vs. 10.6 g/dL; *p* = 0.004). After 3 months, hemoglobin levels increased in both high and low ERI subgroups (high ERI: 9.7 g/dL to 11.1 g/dL, low ERI: 10.6 g/dL to 11.8 g/dL). Hemoglobin levels did not notably decrease before or after the start of hemodialysis in two HIF-PHI-treated patients.

**Conclusions:**

Patients who switched to HIF-PHIs had low hemoglobin levels and high ERI compared with those who continued ESA treatment. Both high ERI and low ERI groups had increased hemoglobin levels after switching.

**Trial registration:**

UMIN Clinical Trials Registry (UMIN000022145, registration date: 30 April 2016).

**Supplementary Information:**

The online version contains supplementary material available at 10.1186/s12882-026-05036-3.

## Background

Anemia is a common complication of chronic kidney disease (CKD), and causes symptoms such as fatigue, heart palpitations, and shortness of breath [1]. In observational studies, anemia in CKD has been associated with adverse clinical outcomes, including cardiovascular events and higher mortality, and may also be associated with faster progression of kidney dysfunction [2–4]. Although anemia management in CKD can improve symptoms and reduce the need for blood transfusions, the extent to which anemia treatment modifies CKD progression or survival outcomes remains uncertain [5].

Findings from large-scale, randomized controlled trials of erythropoiesis-stimulating agents (ESAs), namely, CHOIR [6], CREATE [7], and TREAT [8], have not demonstrated clear kidney or survival benefits and have raised safety concerns at higher achieved hemoglobin levels. Although the emergence of hypoxia-inducible factor-prolyl hydroxylase inhibitors (HIF-PHIs) has expanded the therapeutic options and consistently achieved hemoglobin targets in clinical trials, definitive evidence that these agents improve CKD progression or mortality beyond simple hematologic correction remains to be established.

ESAs are commonly used to treat anemia in CKD; however, some patients may develop resistance to ESA treatment [1]. A multicenter study reported that 13.3% (225/1695) of Japanese patients with non-dialysis CKD had ESA resistance [9], which is linked to poorer outcomes, including increased morbidity and mortality rates and cardiovascular risk [10–14]. Responsiveness to ESAs has been associated with factors such as iron supplements, low C-reactive protein (CRP) levels, low aminoterminal pro-brain natriuretic peptide levels, and low proteinuria [9], while hyporesponsiveness has been associated with increased ferritin and transferrin saturation levels [15]. Prior to undergoing hemodialysis, ESA-treated patients may experience decreased hemoglobin levels due to ESA resistance caused by uremia [16]. Notably, low hemoglobin levels at the start of hemodialysis have been associated with cardiovascular events [17].

HIF-PHIs are oral agents that stimulate endogenous erythropoietin production by stabilizing HIF transcription factors. Treatment with HIF-PHIs has been shown to achieve hemoglobin levels comparable to those achieved with ESAs in patients with CKD-related anemia [18, 19]. Additionally, although overall analyses of randomized controlled trials suggest that HIF-PHIs are noninferior to ESAs with respect to major adverse outcomes, some studies have indicated that certain HIF-PHIs may be associated with a higher risk of major adverse cardiovascular events and other vascular events compared with ESAs, particularly in patients with CKD not receiving dialysis [20]. Large-scale, international phase 3 randomized trials, including the ASCEND program for daprodustat [21, 22] and the PRO_2_TECT and INNO_2_VATE trials for vadadustat [23, 24], have demonstrated that HIF-PHIs are noninferior to ESAs in achieving hemoglobin control across both non-dialysis and dialysis-dependent CKD populations. However, cardiovascular safety profiles have exhibited substantial variability depending on the specific agent and the clinical population studied. In a forest plot stratified by race for major adverse cardiovascular events (MACE; defined as a composite of death from any cause, nonfatal myocardial infarction, or nonfatal stroke), the incidence of MACE was lower among Asian patients in the daprodustat group [21]. The KDIGO 2026 Clinical Practice Guideline for the Management of Anemia in CKD highlights two clinical scenarios in which HIF-PHIs may be considered: (i) ESA hyporesponsiveness or intolerance and (ii) situations in which ESA use is impractical [20]. Unlike ESAs, which require parenteral administration (intravenous or subcutaneous), HIF-PHIs can be administered orally [1], offering an advantage for patients receiving outpatient care, particularly those who do not yet require dialysis. In Japan, the proportion of HIF-PHI use increased from 3.6% in 2020 to 42.7% in 2022 [25].

Importantly, HIF-PHIs are likely to be effective in patients who are resistant to treatment with ESAs [26]. Several studies have investigated their effectiveness in patients resistant to ESAs who are undergoing hemodialysis [27, 28]. However, there have been limited reports regarding the effectiveness of HIF-PHIs in patients resistant to ESAs who do not yet require hemodialysis. The Kidney Disease Improving Global Outcomes (KDIGO) 2026 Clinical Practice Guidelines for the Management of Anemia in Chronic Kidney Disease highlight this gap, noting the lack of studies exploring the effects of HIF-PHIs on hemoglobin levels in these patients [20].

The Reach-J study, a prospective, observational study of Japanese patients with stage G3b to stage G5 CKD [29], provided longitudinal data of Japanese patients before and after the initiation of hemodialysis. Using data from this study, we aimed to explore the characteristics of patients who switched from ESAs to HIF-PHIs. As a secondary objective, we examined changes in hemoglobin levels following switching to HIF-PHIs. We also examined whether HIF-PHIs can maintain hemoglobin levels, which often transiently decline with ESAs before and after the start of hemodialysis.

## Methods

### Study design and patients

The Reach-J study was a nationwide, prospective, observational study of Japanese patients with stage G3b to stage G5 CKD, the design of which has been reported previously [29]. Briefly, eligible patients were selected for enrollment using a predefined random sampling approach at participating sites (as described in detail in [29]) to obtain a representative cohort. The present study analyzed a multicenter, prospective cohort of patients registered in the Reach-J study who were treated with either ESAs or HIF-PHIs. Patients who switched from ESA treatment to HIF-PHIs and those who continued ESA treatment were compared. The date that the first study participant was included was 3 February 2016 and the final observation date was 31 July 2022. Among patients registered in the Reach-J study, all patients who received an ESA during the study period were included, and written informed consent was obtained from all patients. Patients who newly received HIF-PHIs without prior ESA treatment during the study period, and patients who did not receive ESAs or HIF-PHIs during the study period, were excluded.

The study was registered in the UMIN Clinical Trials Registry under the identifier UMIN000022145 (registration date: 30 April 2016) and was conducted in accordance with the Declaration of Helsinki and the Ethical Guidelines for Medical and Health Research Involving Human Subjects (Japanese Ministry of Education, Culture, Sports, Science and Technology and Japanese Ministry of Health, Labour and Welfare, 22 December 2014). The clinical research ethics review committee of each participating medical institution reviewed and approved the implementation plan and consent forms.

### Outcomes

Hemoglobin levels up to 6 months before and 3 months after switching from ESAs to HIF-PHIs, with month 0 as the date of treatment change, were assessed. The first prescription date of HIF-PHIs was defined as day 0.

The patient background variables included age, sex, weight, blood pressure, serum albumin, uric acid, hemoglobin A1c, total cholesterol, low-density lipoprotein cholesterol, high-density lipoprotein cholesterol, triglycerides, hemoglobin, hematocrit, serum iron, unsaturated iron binding capacity, total iron binding capacity (TIBC), ferritin, transferrin, transferrin saturation (TSAT), and CRP. The following were also assessed: estimated glomerular filtration rate (eGFR); urinary protein/creatinine ratio; erythropoietin equivalent dose; erythropoietin resistance index (ERI); type of ESA; type of HIF-PHI (patients who switched treatment only); use of antihypertensive, iron supplement, or statin medications; diabetes mellitus status; and presence of diabetic complications. Diabetes mellitus status and diabetic complications were included as potential factors that may influence anemia management.

The eGFR was calculated as follows: eGFR (mL/min/1.73 m^2^) = 194 × serum creatinine (mg/dL)^−1.094^ × age^− 0.287^ (for men), and eGFR (mL/min/1.73 m^2^) = 194 × serum creatinine (mg/dL)^−1.094^ × age^− 0.287^ × 0.739 (for women). ERI was the erythropoietin equivalent dose per week (IU/week) / body weight (kg) / hemoglobin (g/dL). The erythropoietin equivalent dose per week was calculated by multiplying the weekly dose of darbepoetin alfa and epoetin beta pegol by 200 and 225, respectively.

### Statistical methods

The population for this study included all patients who were in the Reach-J study [29]. Data were summarized by the number of patients, mean, and standard deviation (SD) for continuous data, and by frequencies and proportions for categorical data.

Between-group differences in continuous variables were assessed using Welch’s t-test for two independent groups. Categorical variables were compared using Fisher’s exact test.

For patients who switched from ESAs to HIF-PHIs, the timepoint for comparison was the day closest to the date of initiation of HIF-PHI treatment. For ESA-related indicators (ESA type, erythropoietin equivalent dose, and ERI), the timepoint for comparison was the day on which ESA-related indicators were measured that was closest to the date of initiation of HIF-PHI treatment. For patients who continued ESA treatment, the timepoint for comparison was the time of study enrollment. Patients who continued ESA treatment had received treatment with ESAs for 3 months prior to the time of enrollment. Dose titration of HIF-PHIs following the transition to hemodialysis was not evaluated for the five HIF-PHIs currently used in Japan because of the substantial differences in dosing regimens across these agents, which made a unified assessment of dose adjustment patterns infeasible.

Subgroup analyses were conducted by ERI at the time of HIF-PHI administration, comparing patients with an ERI below the median ERI value (low ERI subgroup) and those with an ERI at or above the median ERI value (high ERI subgroup). The subgroup of patients who initiated hemodialysis was also analyzed.

In this observational study, switching from ESAs to HIF-PHIs was determined by treating physicians in routine practice and was not restricted to patients meeting a predefined ERI threshold for ESA hyporesponsiveness; therefore, the ERI was used descriptively to characterize ESA dose requirements. Because this was an observational study without protocol-mandated visit schedules, hemoglobin measurements were obtained at variable time points in routine practice. The switch date (month 0) was defined as the date of the first HIF-PHI prescription, and the baseline hemoglobin value was defined as the measurement at (or closest to) the switch date. The 3‑month post-switch assessment was defined using a 3‑month follow-up window.

When calculating hemoglobin levels in patients who switched treatment, missing data from 1 to 6 months before and 1–3 months after the switch were supplemented by the last observation carried forward method for up to 3 months. This window was chosen to reflect routine clinical practice in Japan, in which oral medications can be prescribed for up to 3 months and a proportion of patients are followed up at approximately 3‑month intervals.

The significance level was 0.05 and the confidence coefficient was 95%. No adjustment for multiple comparisons was performed. All tests were two-tailed and conducted using SAS version 9.4 (SAS Institute Inc., Cary, NC, USA).

## Results

### Patients

Between February 2016 and July 2022, a total of 34 patients who switched from ESAs to HIF-PHIs and 447 patients who continued ESA treatment were included. The patient background characteristics are summarized in Table 1 and Table S1 in Additional file 1. There were 14 (41.2%) male patients who switched treatment and 251 (56.2%) male patients who continued ESA treatment.

**Table 1:** Patient background characteristics

	Patients who switched to HIF-PHIs (*N* = 34)	Patients who continued with ESAs (*N* = 447)	*p*-value
Male sex, *n* (%)	14 (41.2)	251 (56.2)	0.108
Age, years			
N	34	447	
Mean ± SD	74.4 ± 10.8	71.1 ± 11.9	0.092
BMI, kg/m^2^			
N	34	447	
Mean ± SD	22.9 ± 4.3	23.0 ± 7.0	0.95
Antihypertensive drugs, n (%)	21 (61.8)	391 (87.5)	< 0.001
Diabetes medication, n (%)	6 (17.6)	123 (27.5)	0.236
Iron supplements, n (%)	6 (17.6)	75 (16.8)	0.815
Statins, n (%)	9 (26.5)	166 (37.1)	0.268
Type of ESA, n (%)			
Epoetin beta pegol	23 (67.6)	238 (53.2)	
Darbepoetin alfa	11 (32.4)	209 (46.8)	
Erythropoietin equivalent dose			
N	34	447	
Mean ± SD	27066.2 ± 19476.5	22110.2 ± 16951.9	0.158
ERI			
N	34	447	
Mean ± SD	13.0 ± 10.5	9.7 ± 8.5	0.079
Type of HIF-PHI, n (%)			
Roxadustat	16 (47.1)	-	
Vadadustat	6 (17.6)	-	
Daprodustat	9 (26.5)	-	
Molidustat sodium	1 (2.9)	-	
Enarodustat	2 (5.9)	-	
Diabetes mellitus, n (%)	10 (29.4)	181 (40.7)	0.210
Systolic blood pressure, mmHg			
N	7	299	
Mean ± SD	121.3 ± 18.4	132.9 ± 18.7	0.147
Diastolic blood pressure, mmHg			
N	7	300	
Mean ± SD	60.0 ± 16.7	70.8 ± 12.0	0.137
Hemoglobin, g/dL			
N	34	447	
Mean ± SD	10.0 ± 1.3	10.8 ± 1.2	0.002
Hematocrit, %			
N	15	391	
Mean ± SD	31.0 ± 3.8	33.3 ± 3.9	0.032
eGFR, mL/min/1.73 m^2^			
N	29	402	
Mean ± SD	13.6 ± 7.6	15.4 ± 8.0	0.237
Urine protein/urine creatinine ratio			
N	1	18	
Mean ± SD	1074	9696.5 ± 17033.4	NA
Serum iron, µg/dL			
N	22	185	
Mean ± SD	67.3 ± 26.7	88.3 ± 29.0	0.002
TIBC, µg/dL			
N	22	164	
Mean ± SD	252.3 ± 56.8	265.7 ± 47.1	0.298
UIBC, µg/dL			
N	22	164	
Mean ± SD	185.0 ± 65.3	178.9 ± 54.4	0.680
Ferritin, ng/mL			
N	18	145	
Mean ± SD	251.3 ± 210.9	166.7 ± 171.8	0.118
TSAT, %			
N	22	165	
Mean ± SD	27.9 ± 12.4	34.0 ± 13.0	0.042
CRP, mg/dL			
N	13	241	
Mean ± SD	0.4 ± 0.6	0.4 ± 0.9	0.988

Welch’s t-test for two independent groups was used for continuous variables; Fisher’s exact test was used for categorical variables. As only one patient in the switched group had available data for urine protein/urine creatinine ratio, the variance could not be estimated and the p-value could not be calculated using the Welch’s t-test; thus, we have entered “NA”

BMI, body mass index; CRP, C-reactive protein; eGFR, estimated glomerular filtration rate; ERI, erythropoietin resistance index; ESA, erythropoiesis-stimulating agent; HIF-PHI, hypoxia-inducible factor-prolyl hydroxylase inhibitor; N, number; NA, not available; SD, standard deviation; TIBC, total iron binding capacity; TSAT, transferrin saturation; UIBC, unsaturated iron binding capacity

Among the patients who switched treatment, the mean (SD) age was 74.4 (10.8) years. In patients who continued with ESAs, the mean (SD) age was 71.1 (11.9) years. A lower proportion of patients who switched treatment used antihypertensive drugs than those who continued ESA treatment (61.8% vs. 87.5%; *p* < 0.001).

Patients who switched treatment had numerically higher mean ERI (13.0 vs. 9.7, *p* = 0.079) than those who continued with ESAs. Patients who switched treatment also had a lower mean serum albumin (3.5 g/dL vs. 3.8 g/dL, *p* = 0.015), hemoglobin (10.0 g/dL vs. 10.8 g/dL, *p* = 0.002), hematocrit (31.0% vs. 33.3%, *p* = 0.032), serum iron (67.3 µg/dL vs. 88.3 µg/dL, *p* = 0.002), and TSAT (27.9% vs. 34.0%, *p* = 0.042) compared with those who continued ESA treatment. CRP data were available for only 13 of the 34 patients in the switch group.

### Hemoglobin and other measurements over time

**Fig. 1:**
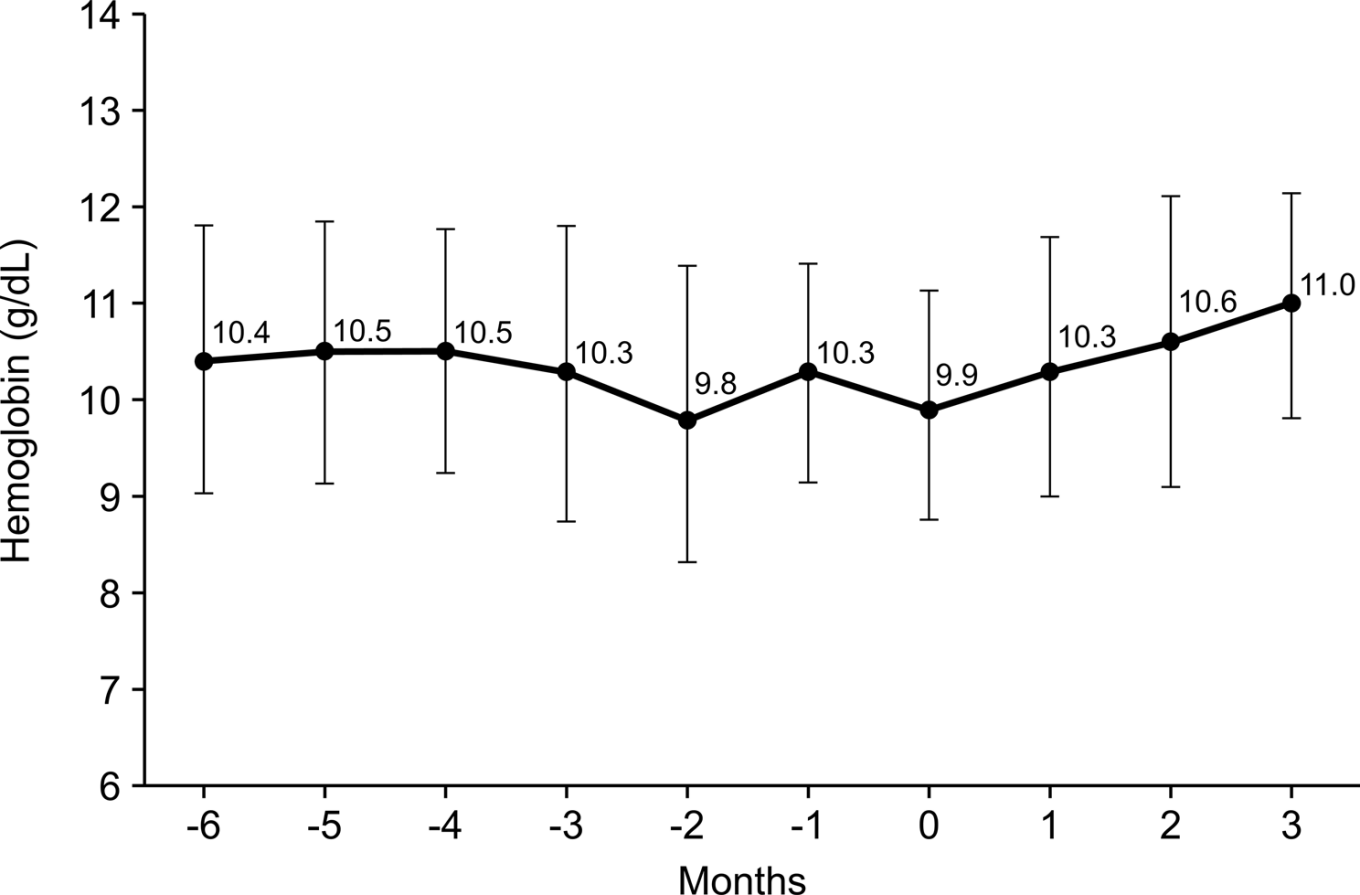
Hemoglobin concentration over time. Data are shown as mean values with standard deviation. At month 0, patients switched from erythropoiesis-stimulating agents to hypoxia-inducible factor-prolyl hydroxylase inhibitors

**Fig. 2:**
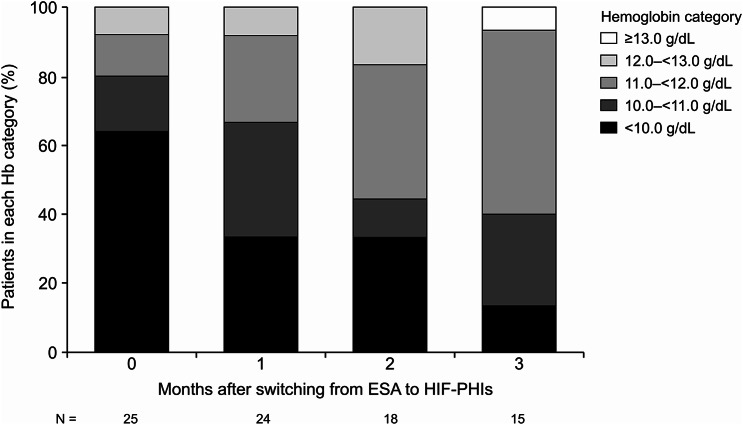
Distribution of patients by hemoglobin category over 3 months after switching from ESA to HIF-PHI treatment. ESA, erythropoiesis-stimulatingagent; Hb, hemoglobin; HIF-PHI, hypoxia-inducible factor–prolyl hydroxylase inhibitor

**Fig. 3:**
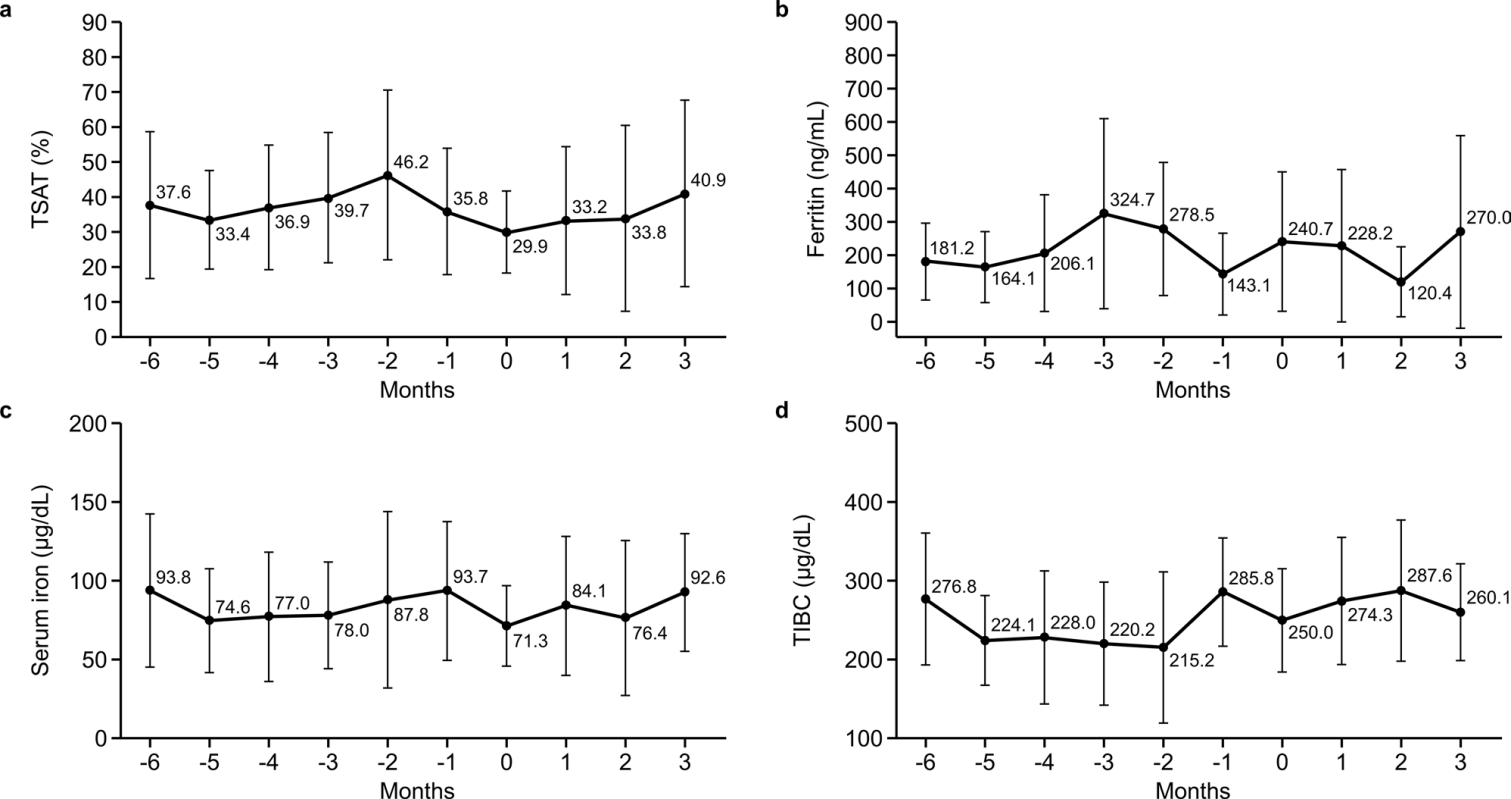
Hematological test results over time. Change over time in (**a**) transferrin saturation, (**b**) ferritin, (**c**) serum iron, and (**d**) total iron binding capacity. Data are shown as mean values with standard deviation. At month 0, patients switched from erythropoiesis-stimulating agents to hypoxia-inducible factor-prolyl hydroxylase inhibitors. TSAT, transferrin saturation; TIBC, total iron binding capacity

Although 34 patients switched from ESAs to HIF-PHIs, only 25 patients had sufficient available data to evaluate outcomes up to 3 months after the switch; therefore, the analyses shown in Figs. 1, 2 and 3; Table 2 are based on these 25 patients. Additionally, of the 25 participants, data was available for 24 patients 1 month after the switch, 18 patients 2 months after the switch, and 15 patients 3 months after the switch. The change over time in hemoglobin levels and the change in hemoglobin categories are shown in Figs. 1 and 2, respectively. Mean hemoglobin levels temporarily decreased prior to switching to HIF-PHIs (9.8 g/dL at 2 months before switching), followed by an increase to 11.0 g/dL (*n* = 15) at 3 months after switching (Fig. 1). The percentage of patients with hemoglobin levels < 10.0 g/dL decreased from 64% (16/25 patients) at month 0 to 13% (2/15 patients) 3 months after switching to HIF-PHIs (Fig. 2).

**Table 2:** Parameters prior to and up to 3 months after switching to HIF-PHIs

	Prior to switching to HIF-PHIs	After switching to HIF-PHIs	*p*-value
Hemoglobin, g/dL			
N	25	25	
Mean ± SD	9.9 ± 1.2	10.6 ± 1.4	0.004
Hemoglobin category, g/dL, n (%)			
< 10.0	16 (64.0)	7 (28.0)	0.016
10.0 to < 11.0	4 (16.0)	6 (24.0)	
11.0 to < 12.0	3 (12.0)	10 (40.0)	
12.0 to < 13.0	2 (8.0)	1 (4.0)	
≥ 13.0	0 (0.0)	1 (4.0)	
Ferritin, ng/mL			
N	12	11	
Mean ± SD	240.7 ± 208.9	262.5 ± 266.1	0.785
TSAT, %			
N	15	11	
Mean ± SD	29.9 ± 11.7	35.7 ± 25.3	0.327
TIBC, µg/dL			
N	15	11	
Mean ± SD	250.0 ± 65.6	280.0 ± 81.9	0.025
Serum iron, µg/dL			
N	15	11	
Mean ± SD	71.3 ± 25.5	84.5 ± 41.7	0.242

Welch’s t-test for two independent groups was used for continuous variables; Fisher’s exact test was used for categorical variables

HIF-PHI, hypoxia-inducible factor-prolyl hydroxylase inhibitor; N, number; SD, standard deviation; TIBC, total iron binding capacity; TSAT, transferrin saturation

Changes over time in iron-related markers are shown in Fig. 3. After switching to HIF-PHIs, TIBC trended to increase from 250.0 µg/dL at month 0 to 260.1 µg/dL at month 3 (Fig. 3d). Comparisons of iron-related markers between the pre-switch and the final observation point 3 months post-switch are shown in Table 2. Mean (SD) hemoglobin levels increased from 9.9 (1.2) g/dL prior to switching to 10.6 (1.4) g/dL after switching (*p* = 0.004) (Table 2). The post-switch values represent the mean ± SD at the last observation within 3 months after switching among the 25 patients with available data at the time of switching. The distribution of hemoglobin categories also changed after switching to HIF-PHI treatment (*p* = 0.016). Mean (SD) TIBC also increased from 250.0 (65.6) µg/dL prior to switching to 280.0 (81.9) µg/dL (*p* = 0.025).

**Fig. 4:**
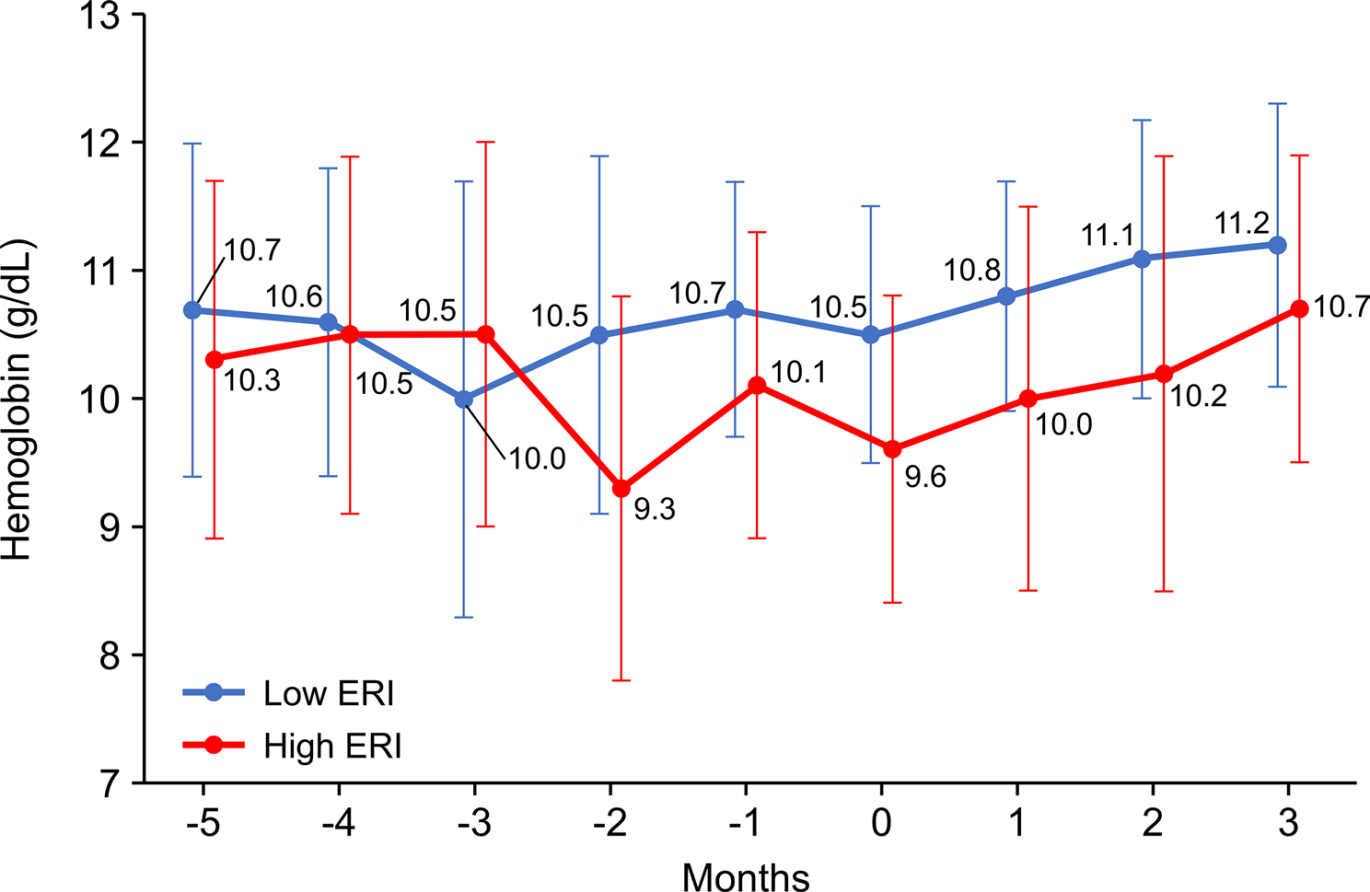
Hemoglobin concentration over time in low and high erythropoietin resistance index subgroups. The low ERI subgroup included patients with an ERI below the median (< 11.7) immediately prior to switching treatment. The high ERI subgroup included patients with an ERI ≥ 11.7 immediately prior to switching treatment. Data are shown as mean values with standard deviation. At month 0, patients switched from erythropoiesis-stimulating agents to hypoxia-inducible factor-prolyl hydroxylase inhibitors. ERI, erythropoietin resistance index

In the high ERI group, after switching to HIF-PHIs, the mean hemoglobin level was 9.7 g/dL at the time of HIF-PHI administration and 11.1 g/dL at 3 months of treatment. In the low ERI group, the mean hemoglobin level was 10.6 g/dL at the time of HIF-PHI administration and 11.8 g/dL at 3 months of treatment (Fig. 4). Patients who continued ESA treatment had a trend of lowered hemoglobin prior to and immediately following hemodialysis (Figure S1 in Additional file 1). Two patients received HIF-PHIs continuously until the start of hemodialysis (patient 1 and patient 2), and there was no notable decrease in hemoglobin levels prior to or after the start of hemodialysis (month 0) for either of these patients.

## Discussion

Based on data from the observational Reach-J study [29], this study clarified the characteristics of patients who switched from ESAs to HIF-PHIs and those who continued treatment with ESAs. Notably, we found that patients who switched from ESAs to HIF-PHIs had numerically higher ERI values and lower hemoglobin levels than patients who continued treatment with ESAs. Prior to switching treatment, hemoglobin levels declined, but this was followed by an increase in hemoglobin levels after switching to HIF-PHIs. Additionally, mean hemoglobin levels and mean TIBC increased after switching to HIF-PHIs.

Importantly, the proportion of patients with hemoglobin levels below 10.0 g/dL decreased following switching to HIF-PHIs. Hemoglobin levels increased in both patients with high ERI and those with low ERI who switched to HIF-PHIs, suggesting that switching to HIF-PHIs may be effective at increasing hemoglobin levels across a range of ESA responsiveness.

In our study, nearly half of the patients who switched to HIF-PHIs received roxadustat (*n* = 16, 47.1%), and the second most common HIF-PHI was daprodustat (*n* = 9, 26.5%). To the best of our knowledge, few studies have investigated the effects of switching from ESAs to HIF-PHIs in patients with non-dialysis CKD in routine clinical practice. However, several studies have evaluated individual HIF-PHIs in patients undergoing dialysis. A study of the HIF-PHI roxadustat in 31 patients undergoing hemodialysis treatment who were resistant to ESAs [30] reported that TIBC increased and that TSAT decreased. This is generally consistent with our observed results that TIBC increased in patients following switching to HIF-PHIs. This likely reflects an increase in transferrin following switching to HIF-PHIs [31, 32], which may improve iron transport and availability for erythropoiesis. Additionally, HIF-PHIs may increase iron utilization by suppressing hepcidin and thereby promoting intestinal iron absorption and iron mobilization [31, 32]. Furthermore, in both non-dialysis and dialysis populations, roxadustat dose requirements did not increase in patients with higher baseline high-sensitivity C-reactive protein levels [33]. In a study evaluating the efficacy of daprodustat in 271 dialysis patients, hemoglobin levels were maintained with 6 mg of daprodustat even in the ERI subgroup (≥ 8.0 IU/kg per week per g/dL) [32]. Overall, these findings emphasize the importance of monitoring hemoglobin response after initiating HIF-PHI therapy to guide subsequent dose adjustment, as is standard practice for anemia treatments.

In this cohort, hemoglobin levels remained stable during the transition to hemodialysis in the two patients who received HIF-PHIs, in contrast with the declining trends often observed with ESA therapy during the onset of kidney failure [34, 35]. While these observations are limited by the small sample size, they support the hypothesis that HIF-PHIs may mitigate the impact of uremia-associated ESA resistance. The widespread inflammatory response characteristic of uremia [36] may have a smaller inhibitory impact on HIF-PHIs, which are thought to exert their anemia-improving effects regardless of inflammatory responses [37]. Further large-scale studies are warranted to determine if switching to HIF-PHIs can consistently stabilize hemoglobin levels during the peri-dialysis period.

A study of daprodustat in seven patients undergoing hemodialysis treatment with high ERI [38] reported that two patients (29%) had increased hemoglobin following 16 weeks of daprodustat. This aligns with the present finding that the mean hemoglobin levels in patients with high ERI increased from 9.7 g/dL to 11.1 g/dL at 3 months.

Other studies have indicated that HIF-PHIs may be effective in patients with hyporesponsiveness to ESAs. One study reported that the addition of roxadustat to ESA treatment corrected hyporesponsiveness in all nine patients undergoing peritoneal dialysis [39]. Similarly, a recent retrospective study found that patients with ESA hyporesponsiveness undergoing peritoneal dialysis who were treated with roxadustat had increased mean hemoglobin levels by week 16 (89.8 g/L vs. 118 g/L; *p* < 0.05), whereas ESA-treated patients did not [40]. Another recent study reported that roxadustat-treated patients with hyporesponsiveness to ESAs undergoing hemodialysis had improvements in hemoglobin levels and iron metabolism [27]. Moreover, a recent retrospective study (*N* = 55) reported that patients with hyporesponsiveness to ESAs undergoing hemodialysis who switched to roxadustat had increased hemoglobin levels [28].

The 2026 KDIGO Clinical Practice Guideline for the Management of Anemia in CKD [20] identifies HIF-PHIs as effective oral alternatives to ESAs, capable of achieving comparable hemoglobin targets through the stimulation of endogenous erythropoietin production. Nevertheless, the guidelines maintain a preference for ESAs when escalating therapy—contingent on no other correctable causes of anemia—primarily because of their extensive long-term safety profile. KDIGO underscores that the clinical benefits of HIF-PHIs in hyporesponsive patients (specifically regarding outcomes beyond hematologic correction) remain unproven, necessitating a shared decision-making approach [20]. In this context, our findings provide preliminary real‑world data on patients who switched from ESAs to HIF-PHIs, many of whom had higher ERI and lower hemoglobin levels than those who continued ESA therapy.

We acknowledge several limitations of this study. First, only 34 of 481 ESA-treated patients (7.1%) were switched to HIF-PHIs during the observation period, and the switch group was much smaller than the ESA continuation group (*n* = 34 vs. *n* = 447), resulting in a substantial imbalance in sample size. This marked disparity may have reduced the statistical power and precision of between-group comparisons and increased the risk of type II error; therefore, the comparative findings should be interpreted with caution and considered exploratory. Second, follow-up data extending to 3 months after the switch were available for only 25 of these 34 patients, which may have introduced selection bias. Third, only two patients initiated hemodialysis after switching from ESAs to HIF-PHIs, precluding any meaningful assessment of hemoglobin trajectories around dialysis initiation. Fourth, as this was an observational study, residual confounding and confounding by indication cannot be excluded. Fifth, CRP values were available for only 13 of 34 patients in the switch group, which limited our ability to examine inflammation in relation to ERI subgroups or hemoglobin response after switching. In addition, laboratory measurements were performed as part of routine clinical practice rather than according to a predefined schedule. Consequently, data were not collected at uniform time points across all patients, and both the number and characteristics of patients contributing data varied at each time point. This may have introduced variability in patient characteristics and contributed to fluctuations in the observed temporal trends. Therefore, changes based on descriptive mean values over time should be interpreted with caution. Furthermore, visualization of individual patient trajectories (e.g., spaghetti plots) may help complement the interpretation of population-level trends and is considered a subject for future investigation. Finally, generalizability may be limited: only Japanese patients were included, and the availability and uptake of HIF-PHIs for anemia in non‑dialysis CKD vary across countries/regions. Consequently, real‑world evidence regarding HIF-PHIs in non‑dialysis CKD, particularly in patients with ESA hyporesponsiveness, has been reported predominantly from Japan and China, whereas in the United States, HIF-PHIs are currently approved only for dialysis‑dependent CKD, limiting the availability of non‑dialysis CKD real‑world data [37].

In conclusion, this study found that patients who switched from ESAs to HIF-PHIs had low hemoglobin levels and tended to have high ERI. Additionally, patients with high ERI levels and those with low ERI levels both showed an increase in hemoglobin levels following switching to HIF-PHIs. Larger studies with longer follow-up are warranted to confirm these findings.

## Supplementary Information

Below is the link to the electronic supplementary material.

Supplementary Material 1

## Data Availability

The datasets used in the current analysis are available from the corresponding author upon reasonable request.

## Abbreviations

CKD: Chronic kidney disease
ESA: erythropoiesis-stimulating agent
CRP: C-reactive protein
HIF-PHI: Hypoxia-inducible factor-prolyl hydroxylase inhibitor
KDIGO: Kidney disease improving global outcomes
TIBC: Total iron binding capacity
TSAT: Transferrin saturation
eGFR: Estimated glomerular filtration rate
ERI: Erythropoietin resistance index
SD: Standard deviation
