# Supplementary material for: Patient characteristics and treatment efficacy after switching to hypoxia-inducible factor-prolyl hydroxylase inhibitors from erythropoiesis-stimulating agents in non-dialysis-dependent chronic kidney disease patients: the Reach-J CKD cohort study
Source: BMC Nephrol. 2026 May 25;27:434. doi: 10.1186/s12882-026-05036-3 (PMC13383514; doi:10.1186/s12882-026-05036-3)

**Additional Information**

**Table S1.** Additional patient background characteristics**.**

|  | **Patients who switched to HIF**–**PHIs**  **(N = 34)** | **Patients who continued with ESAs**  **(N = 447)** | **p-value** |
| --- | --- | --- | --- |
| Serum albumin, g/dL |  |  |  |
| N | 15 | 377 |  |
| Mean ± SD | 3.5 ± 0.4 | 3.8 ± 0.5 | 0.015 |
| Uric acid, mg/dL |  |  |  |
| N | 15 | 383 |  |
| Mean ± SD | 6.3 ± 1.8 | 6.1 ± 1.3 | 0.717 |
| HbA1c, % |  |  |  |
| N | 7 | 172 |  |
| Mean ± SD | 6.2 ± 0.6 | 6.1 ± 0.8 | 0.727 |
| Total cholesterol, mg/dL |  |  |  |
| N | 11 | 254 |  |
| Mean ± SD | 189.9 ± 51.0 | 175.8 ± 36.0 | 0.385 |
| LDL cholesterol, mg/dL |  |  |  |
| N | 12 | 237 |  |
| Mean ± SD | 78.6 ± 30.8 | 95.0 ± 28.8 | 0.096 |
| HDL cholesterol, mg/dL |  |  |  |
| N | 14 | 266 |  |
| Mean ± SD | 55.0 ± 17.8 | 53.3 ± 17.5 | 0.745 |
| Triglycerides, mg/dL |  |  |  |
| N | 13 | 287 |  |
| Mean ± SD | 147.7 ± 71.8 | 128.5 ± 65.4 | 0.361 |

Welch’s t-test for two independent groups was used for continuous variables; Fisher’s exact test was used for categorical variables.

HDL, high-density lipoprotein; LDL, low-density lipoprotein; N, number; SD, standard deviation.

**Figure S1.** Hemoglobin concentration over time in the hemodialysis subgroup

Data are shown as mean values with standard deviation. At month 0, patients began hemodialysis treatment. At month −2, patient 1 switched to HIF–PHIs. At month −5, patient 2 switched to HIF–PHIs.

ESA, erythropoiesis-stimulating agent; HIF–PHI, hypoxia-inducible factor–prolyl hydroxylase inhibitor.


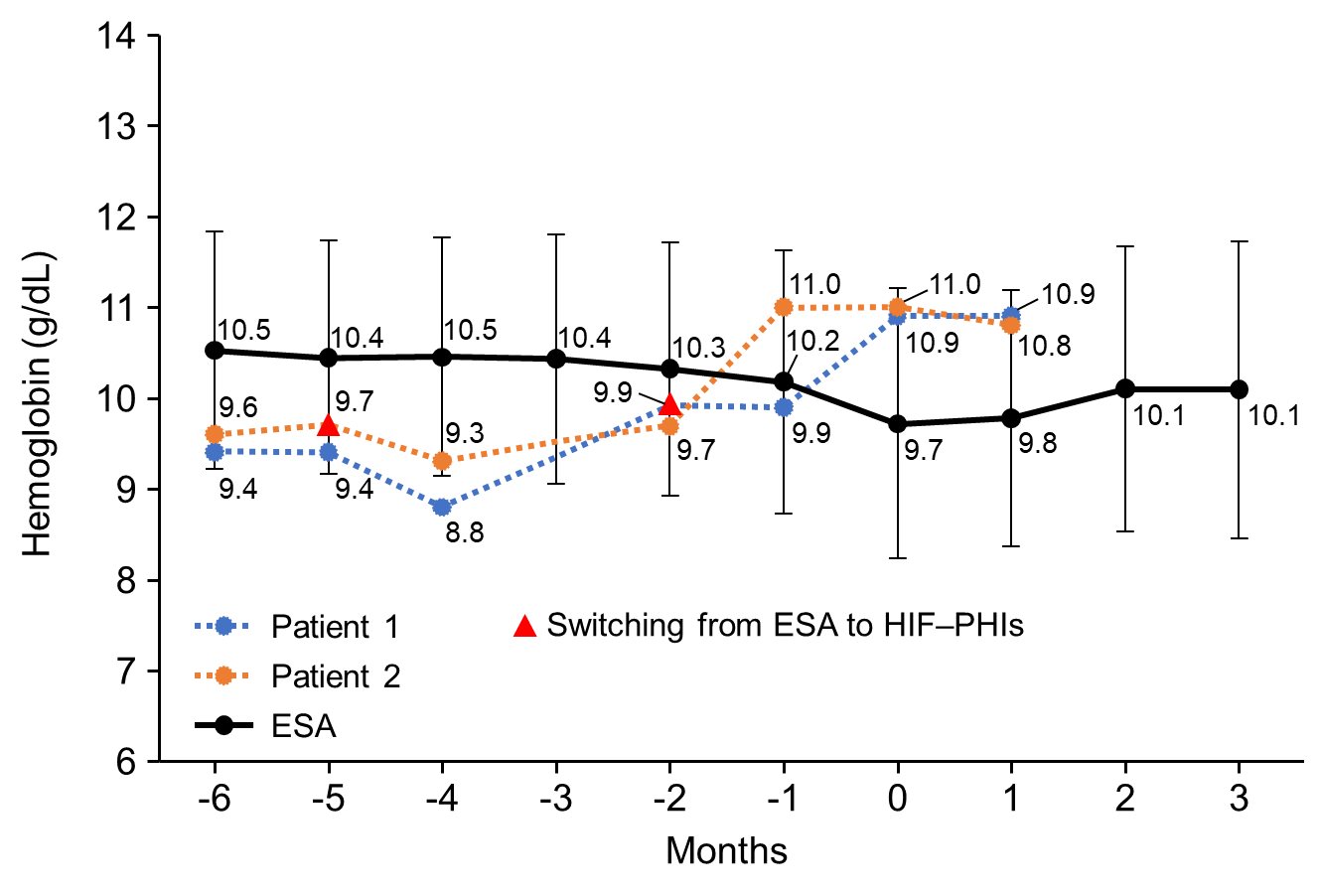

Supplement: Supplementary file 1 — Supplementary Material 1 [file 12882_2026_5036_MOESM1_ESM.docx]
